# Characterization of novel macrophage cell lines derived from goat and sheep blood and their susceptibility to ruminant virus infections

**DOI:** 10.1128/spectrum.03540-25

**Published:** 2026-05-11

**Authors:** Rina Ikeda, Misako Konishi, Tohru Yanase, Hiroki Tatsumi, Katsunori Murota, Takato Takenouchi

**Affiliations:** 1 Kagoshima Research Station, National Agriculture and Food Research Organization, National Institute of Animal Health, Kagoshima, Japan; 2 Graduate School of Science and Technology, University of Tsukuba, Tsukuba, Ibaraki, Japan; 3 National Agriculture and Food Research Organization, Institute of Agrobiological Sciences, Tsukuba, Japan; National Microbiology Laboratory, Winnipeg, Manitoba, Canada

**Keywords:** goat, macrophages, immortalization, *In vitro *model, ruminant viruses, sheep

## Abstract

Macrophages are present in all vertebrate tissues and play a key role in both the prevention and pathogenesis of infectious diseases. *In vitro* cultures of macrophages provide a valuable tool for studying the innate immune functions of macrophages and macrophage-pathogen interactions in specific animal species. We previously demonstrated that porcine macrophages were effectively immortalized by transferring the simian virus 40 large T antigen (SV40LT) gene and porcine telomerase reverse transcriptase (pTERT) gene using lentiviral vectors. In the present study, we investigated whether this immortalization protocol may be applied to macrophages from small ruminants. Primary goat and sheep macrophages were successfully isolated from mixed cultures of heparinized whole blood with porcine kidney-derived feeder cells. Goat blood-derived macrophages (GBMs) and ovine blood-derived macrophages (OBMs) were then immortalized by introducing the SV40LT and pTERT genes, and immortalized GBM (iGBM) and OBM (iOBM) cell lines were established. iGBM and iOBM cells exhibited typical Iba1-, CD172a-, and CD204-positive macrophage-like phenotypes and the upregulated phosphorylation of nuclear factor-kB, p38, and p44/42 mitogen-activated protein kinases in response to the bacterial cell wall components, lipopolysaccharides, and muramyl dipeptides. These cells retained typical macrophage functions, such as the production of inflammatory cytokines and phagocytosis. iGBM and iOBM cells were susceptible to infection not only by caprine arthritis encephalitis and bluetongue viruses, which primarily affect goats and sheep, but also by arthropod-borne viruses that cause encephalitis primarily in cattle. Therefore, the iGBM and iOBM cell lines provide unique models for investigating the characteristics and pathogenesis of ruminant viruses.

**IMPORTANCE:**

Infectious diseases in goats and sheep cause severe economic losses; therefore, the development of infection prevention and control protocols is important. Since these animals share a number of pathogens with other livestock and humans, making them potential sources of zoonotic diseases, basic research is being conducted to further characterize the pathogens and develop more effective vaccines. Against this background, the significance of the present study lies in establishing new sustainable cell lines from goat and sheep macrophages. These cells retained typical macrophage functions, such as the production of inflammatory cytokines and phagocytosis. Interestingly, they were susceptible to infection not only by caprine arthritis encephalitis and bluetongue viruses, which primarily affect goats and sheep, but also by arthropod-borne viruses that cause encephalitis primarily in cattle. Therefore, the goat and sheep macrophage cell lines established in this study provide practical and reliable models for investigating the host-pathogen interactions of ruminant viruses.

## INTRODUCTION

Goats and sheep are the first ruminants that were domesticated by humans. They have a more suitable body size for domestication, are easier to manage, reach puberty and maturity earlier, have higher reproductive rates, and are more sociable and docile than cattle (1). Goats and sheep remain important livestock around the world for producing meat, milk, fur, and leather (1). Goat and sheep farming has a significant economic impact, as they are essential for the survival of some populations worldwide, particularly in developing countries and arid regions.

Given this background, the development of prevention and control protocols for infectious diseases in goats and sheep is important because they cause serious economic losses (2). Since the increased costs associated with vaccination may also affect goat and sheep farming, basic research is currently underway to further characterize the pathogens and develop more effective vaccines. Goats and sheep share a number of pathogens with other livestock and humans, making them potential sources of zoonotic diseases (3). A comprehensive database on goat and sheep pathogens has recently been developed to provide technical support for research on and the prevention and control of infectious diseases in goats and sheep (2).

Macrophages are representative innate immune cells that are present in all vertebrate tissues and have multiple functions, such as phagocytosis, cytokine production, and antigen presentation (4). They are highly heterogeneous, plastic cells that do not fit into a strict, fully characterized classification system (4). Various viral and bacterial pathogens target macrophages during infection and utilize them to propagate and spread within the host body, thereby evading host defense systems (5). Therefore, *in vitro* cultures of macrophages will provide a useful model for investigating host-pathogen interactions. For example, porcine macrophage cultures are frequently used to isolate and propagate swine viral pathogens, such as porcine reproductive and respiratory syndrome virus and African swine fever virus (6, 7).

Small ruminant lentiviruses (SRLV) primarily infect monocyte/macrophage lineage cells, causing a long-lasting infection that affects the body condition, production, and welfare of goats and sheep (8). Based on these findings, goat and sheep macrophages are expected to become a valuable tool for evaluating the interactions of SRLV, such as caprine arthritis encephalitis virus (CAEV) and maedi-visna virus, with host immune cells. However, collecting primary macrophages from blood samples using traditional methods is inconvenient for *in vitro* analysis due to their low proliferation capacity.

In the present study, we isolated goat blood-derived macrophages (GBMs) and ovine blood-derived macrophages (OBMs) using a mixed primary culture of goat and sheep peripheral blood with porcine kidney-derived feeder cells using the protocol for porcine macrophages described in our previous study (9). Then, we aimed to establish new sustainable immortalized GBM (iGBM) and OBM (iOBM) cell lines by immortalizing GBMs and OBMs, respectively, and to develop methods to use iGBM and iOBM cells as tools for characterizing various ruminant viruses.

## RESULTS

### Isolation and immortalization of GBMs and OBMs

**Fig 1:**
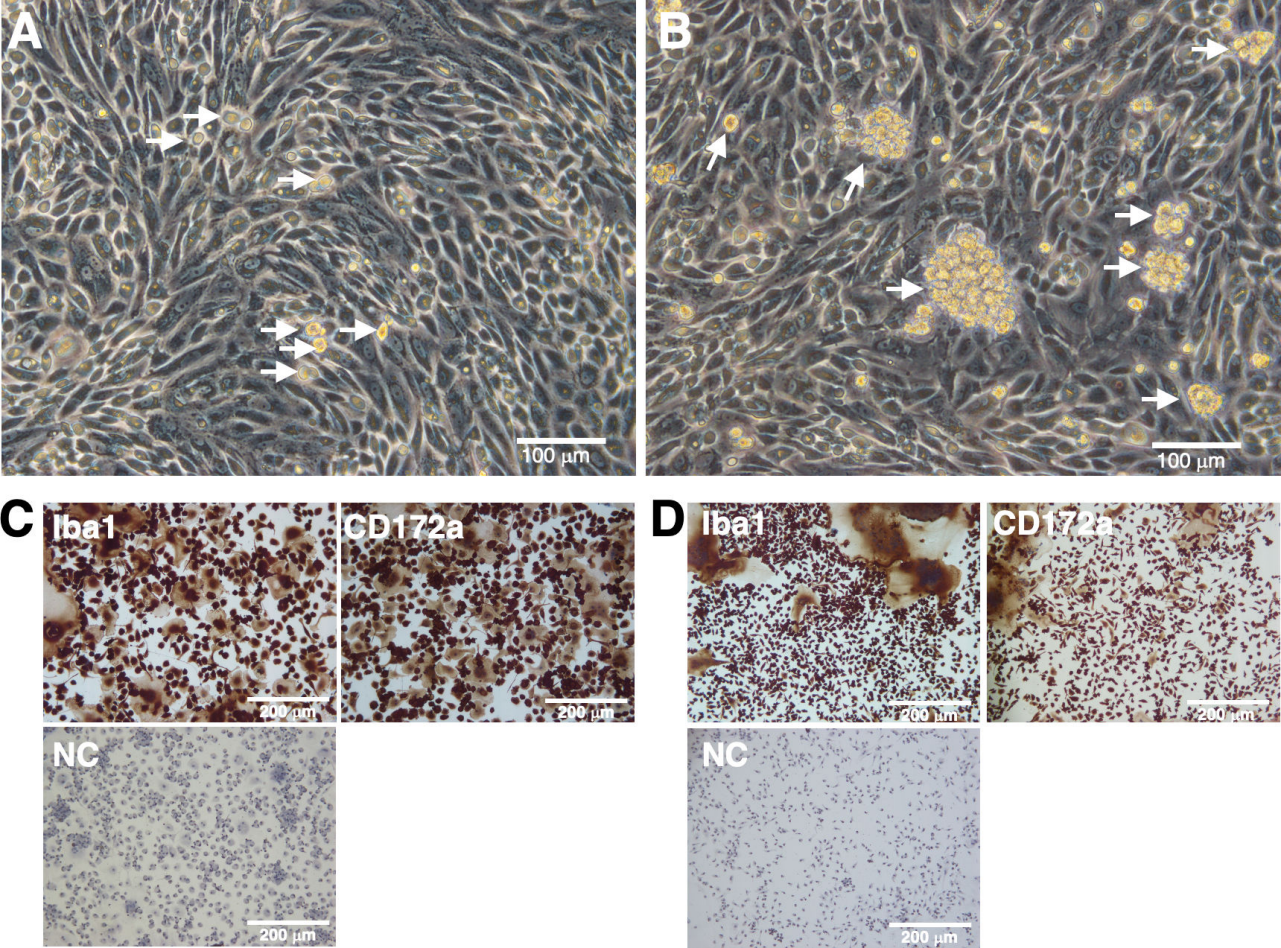
Mixed culture of goat or sheep whole blood with primary porcine kidney feeder cells and the characterization of GBMs and OBMs. GBMs and OBMs proliferated on the primary porcine kidney feeder cell sheet (**A and B**, white arrows). Isolated GBMs and OBMs were seeded on eight-well chamber slides and cultured for 1 day. Cells were then fixed using 4% paraformaldehyde phosphate buffer solution and immunostained with specific antibodies against cell markers of macrophages (Iba1 and CD172a) (*brown*) (**C and D**). No specific staining was observed when cells were treated without primary antibodies (NC: negative control in C and D). All nuclei were counterstained with hematoxylin (*blue*), and the formation of MGCs was observed (**C and D**).

GBMs and OBMs grown under the mixed culture conditions of goat or sheep whole blood with primary porcine kidney feeder cells exhibited a spherical shape and were loosely attached to the feeder cell sheet (Fig. 1A and B). GBMs and OBMs collected from the culture supernatant by centrifugation also exhibited the ability to adhere to non-tissue culture-grade Petri dishes (NTC dishes). Therefore, they were separated from other cell types based on this characteristic. The isolated cells prominently expressed the typical macrophage markers, such as ionized calcium-binding adaptor molecule 1 (Iba1) and CD172a (Fig. 1C and D). The spontaneous formation of multinucleated giant cells (MGCs), characterized by the fusion of primary macrophages, was frequently observed (Fig. 1C and D).

**Fig 2:**
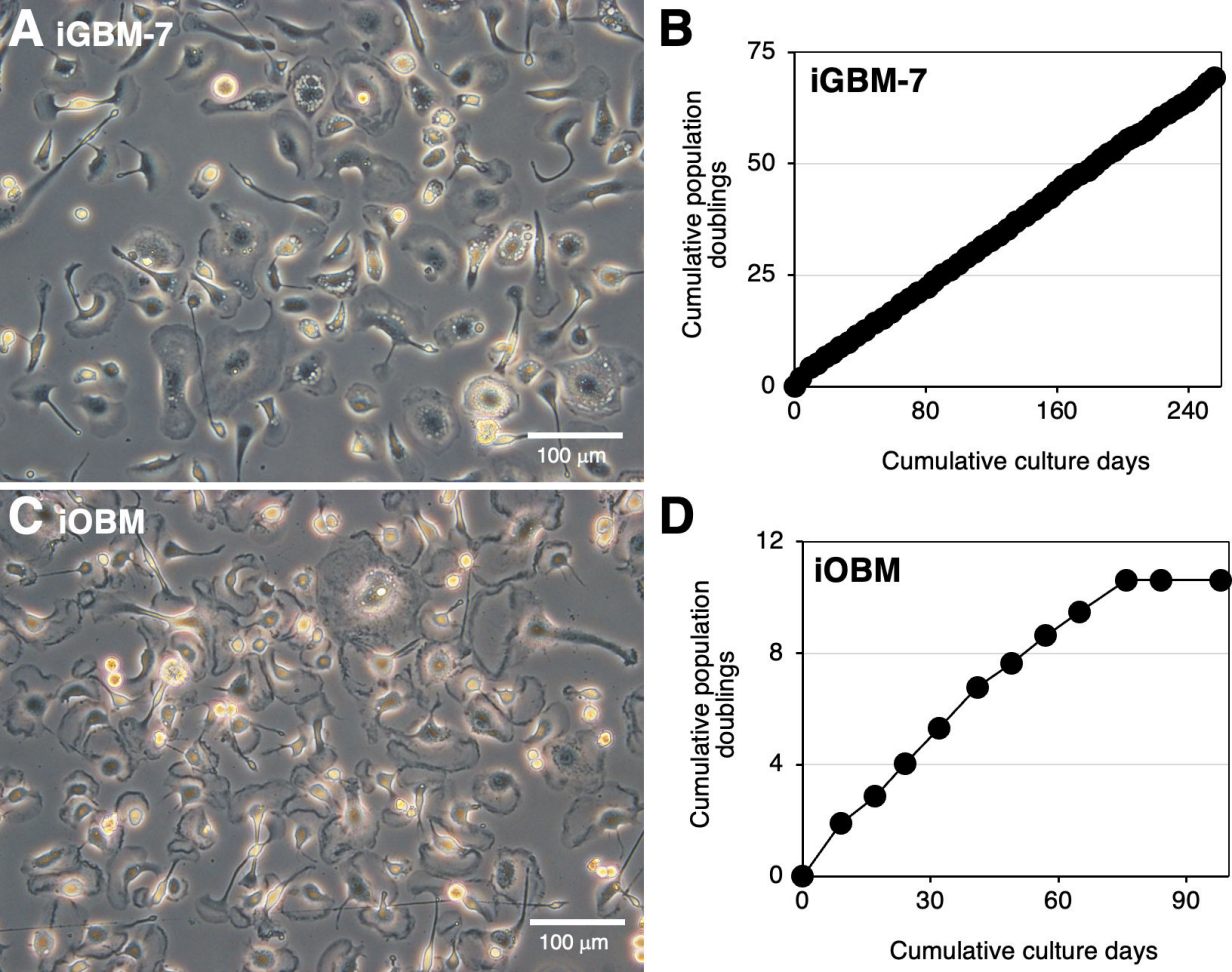
Proliferative potential of iGBM-7 and iOBM cells. The morphologies of iGBM-7 (**A**) or iOBM (**C**) cells were observed under a phase-contrast microscope. The cumulative population doublings of iGBM-7 (**B**) or iOBM (**D**) cells were plotted against the duration of the culture period (in days).

They were then immortalized by transfecting both the SV40 large T antigen (SV40LT) gene and porcine telomerase reverse transcriptase (pTERT) gene using lentiviral vectors to generate the continuously proliferating cell lines, iGBM and iOBM. Several cell lines of iGBM cells were obtained from GBMs, among which iGBM-7 cells were established as a well-growing cell line (Fig. 2A). iGBM-7 cells stably proliferated up to at least 69 population doublings within 256 days (Fig. 2B). Regarding OBMs, one cell line of iOBM was obtained; however, its proliferation nearly stopped at approximately 10 population doublings within 76 days (Fig. 2D). Both cell lines exhibited a typical macrophage-like morphology with ruffled membranes and cell processes (Fig. 2A and C). These cell lines were not contaminated with bovine viral diarrhea virus (BVDV) or CAEV, as confirmed by polymerase chain reactions (PCR) (data not shown).

### Characterization of iGBM-7 and iOBM cells

**Fig 3:**
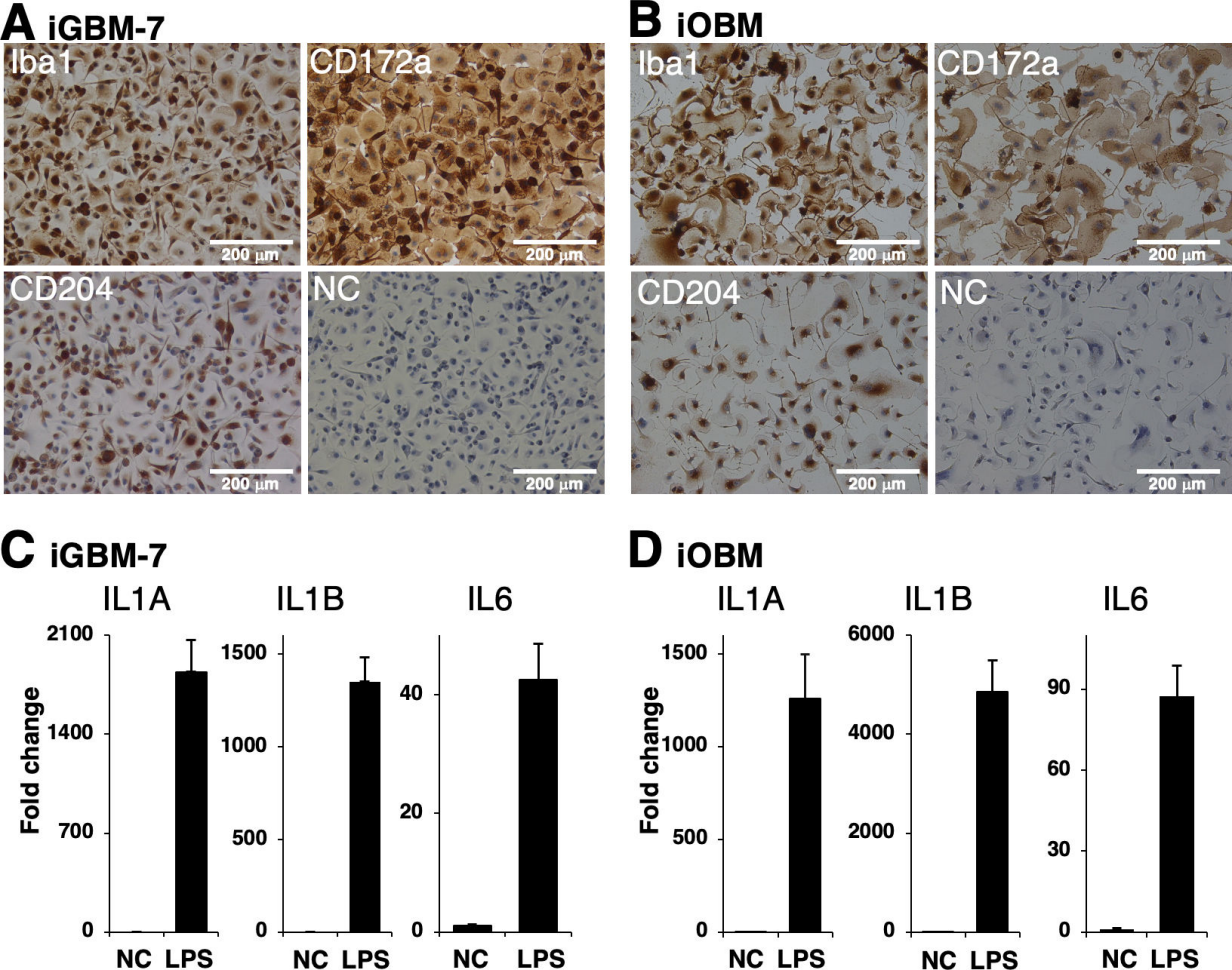
Characterization of iGBM-7 and iOBM cells. Cells were seeded on eight-well chamber slides and cultured for 1 day. iGBM-7 (**A**) or iOBM (**B**) cells were then immunostained with specific antibodies against cell markers of macrophages (Iba1, CD172a, and CD204) (*brown*). No specific staining was observed when cells were treated without primary antibodies (NC: negative control in A and B). All nuclei were counterstained with hematoxylin (*blue*) (**A and B**). Total RNA was recovered from iGBM-7 (**C**) or iOBM (**D**) cells treated with or without 1 µg/mL LPS, and RT-qPCR experiments were performed independently three (**C**) or two (**D**) times. Fold changes from untreated control cells (NC) were expressed as the mean ± SEM.

As shown in Fig. 3, iGBM-7 and iOBM cells were positive for typical macrophage markers (Iba1, CD172a, and CD204) (Fig. 3A and B). Quantitative real-time reverse transcriptase-PCR (qRT-PCR) showed that the stimulation of these cells with lipopolysaccharide (LPS) for 3 h markedly increased the expression of interleukin (IL)-1 family cytokine mRNAs (IL1A and IL1B) (Fig. 3C and D). The mRNA expression of the pro-inflammatory cytokine IL-6 also increased in response to LPS (Fig. 3C and D). The expression level of glyceraldehyde-3-phosphate dehydrogenase (GAPDH) mRNA remained stable with or without the LPS stimulation (data not shown).

### Establishment of iOBM-2 cells and the identification of species in iGBM-7 and iOBM-2 cells

**Fig 4:**
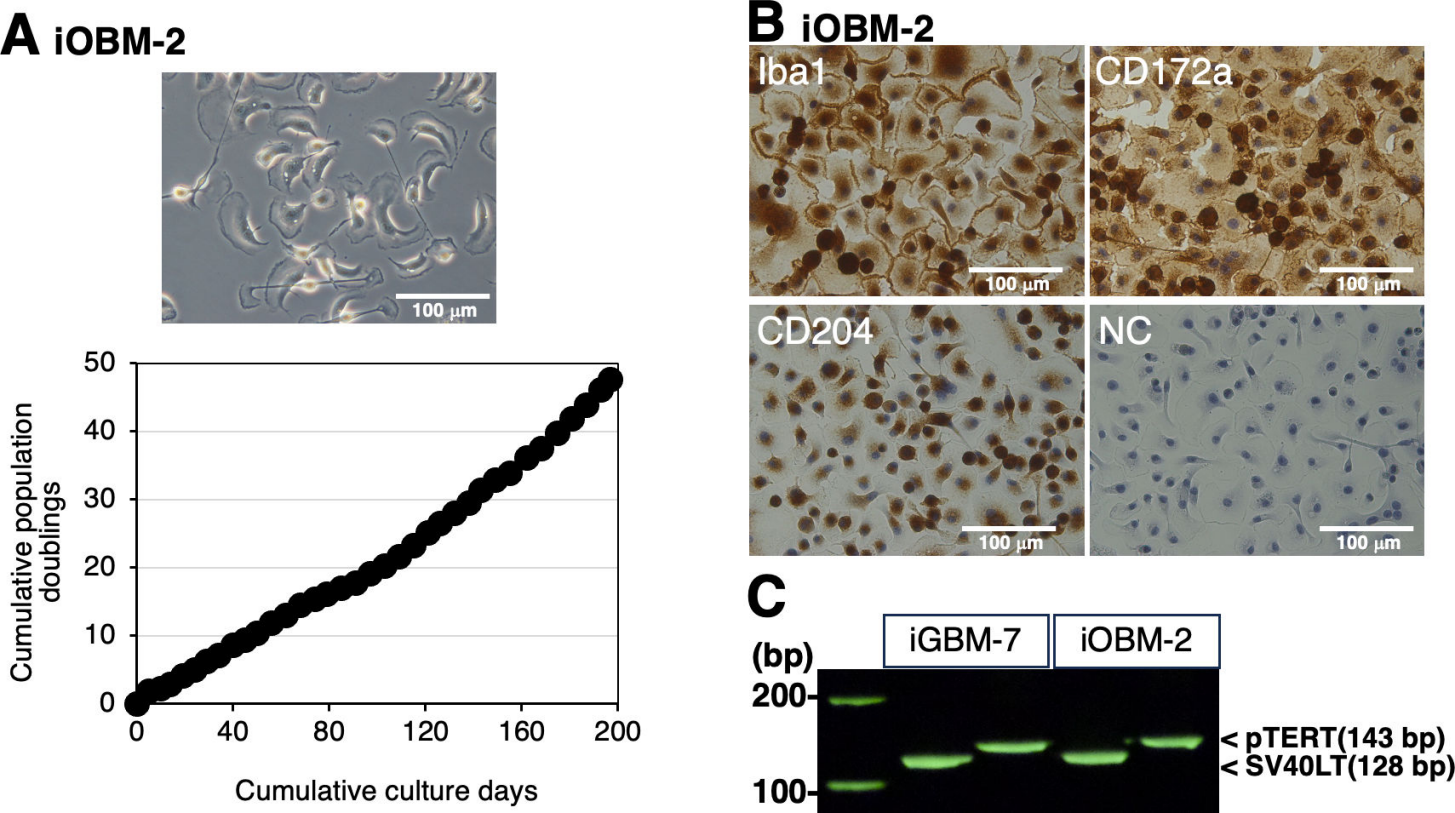
Proliferative potential and characterization of iOBM-2 cells. The morphology of iGBM-2 cells was observed under a phase-contrast microscope (**A**). The cumulative population doublings of iGBM-2 cells were plotted against the duration of the culture period (in days) (**A**). iGBM-2 cells were seeded on eight-well chamber slides, cultured for 1 day, and then immunostained with specific antibodies against cell markers of macrophages (Iba1, CD172a, and CD204) (*brown*) (**B**). No specific staining was observed when cells were treated without primary antibodies (NC: negative control in B). All nuclei were counterstained with hematoxylin (*blue*) (**B**). The transduction of SV40LT and pTERT genes in iOBM-2 and iGBM-7 cells was confirmed by a genomic DNA PCR analysis (**C**).

To improve the proliferative ability of iOBM cells, the transduction of the SV40LT and pTERT genes using a lentiviral vector was additionally applied to these cells. The iOBM-2 cell line was then established as a subline derived from iOBM cells (Fig. 4A). iOBM-2 cells stably proliferated for at least 47 population doublings within 197 days (Fig. 4A) and expressed macrophage markers (Iba1, CD172a, and CD204) (Fig. 4B). Genomic DNA PCR analysis confirmed that the immortalization genes were introduced into both iOBM-2 and iGBM-7 cells (Fig. 4C). In addition, iGBM-7 and iOBM-2 cells were confirmed to be derived from goats (*Capra hircus*) and sheep (*Ovis aries*), respectively, based on partial DNA sequencing of the mitochondrial 12S ribosomal RNA gene (data not shown).

### Phagocytotic activity of and cytoplasmic signal transduction in iGBM-7 and iOBM-2 cells

**Fig 5:**
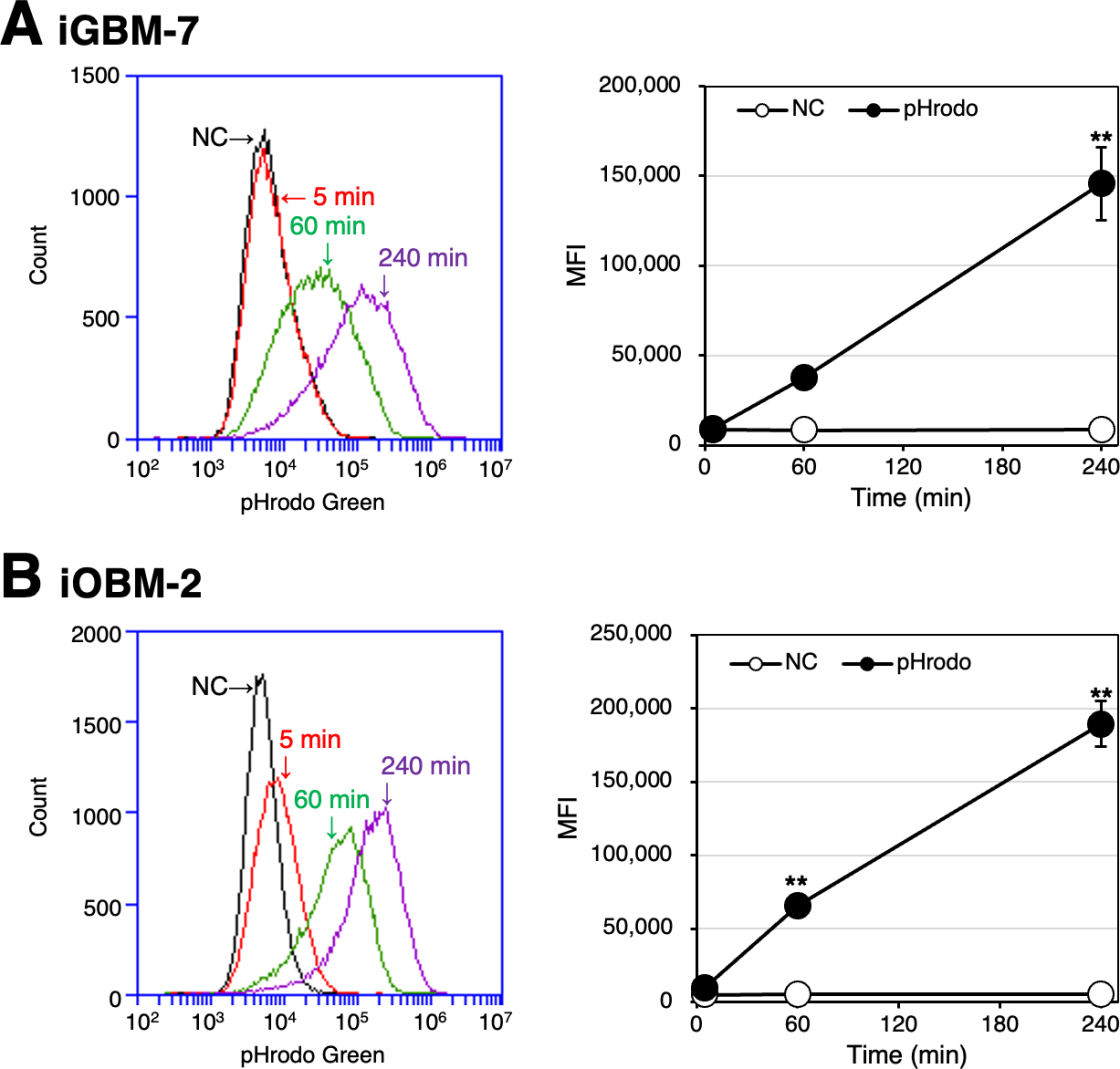
Phagocytotic activity of iGBM-7 and iOBM-2 cells. iGBM-7 (**A**) or iOBM-2 (**B**) cells were treated with pHrodo Green-labeled *E. coli* BioParticles. After 5-min (*red line*), 60-min (*green line*), and 240-min (*purple line*) incubations, the cells were detached and analyzed using a flow cytometer (A and B, histograms). Three independent experiments were performed, and MFI data are expressed as the mean ± SEM (A and B, graphs) (**P < 0.01 vs. 5 min). Cells not treated with pHrodo Green-labeled *E. coli* BioParticles were used as a negative control (NC) (**A and B**).

**Fig 6:**
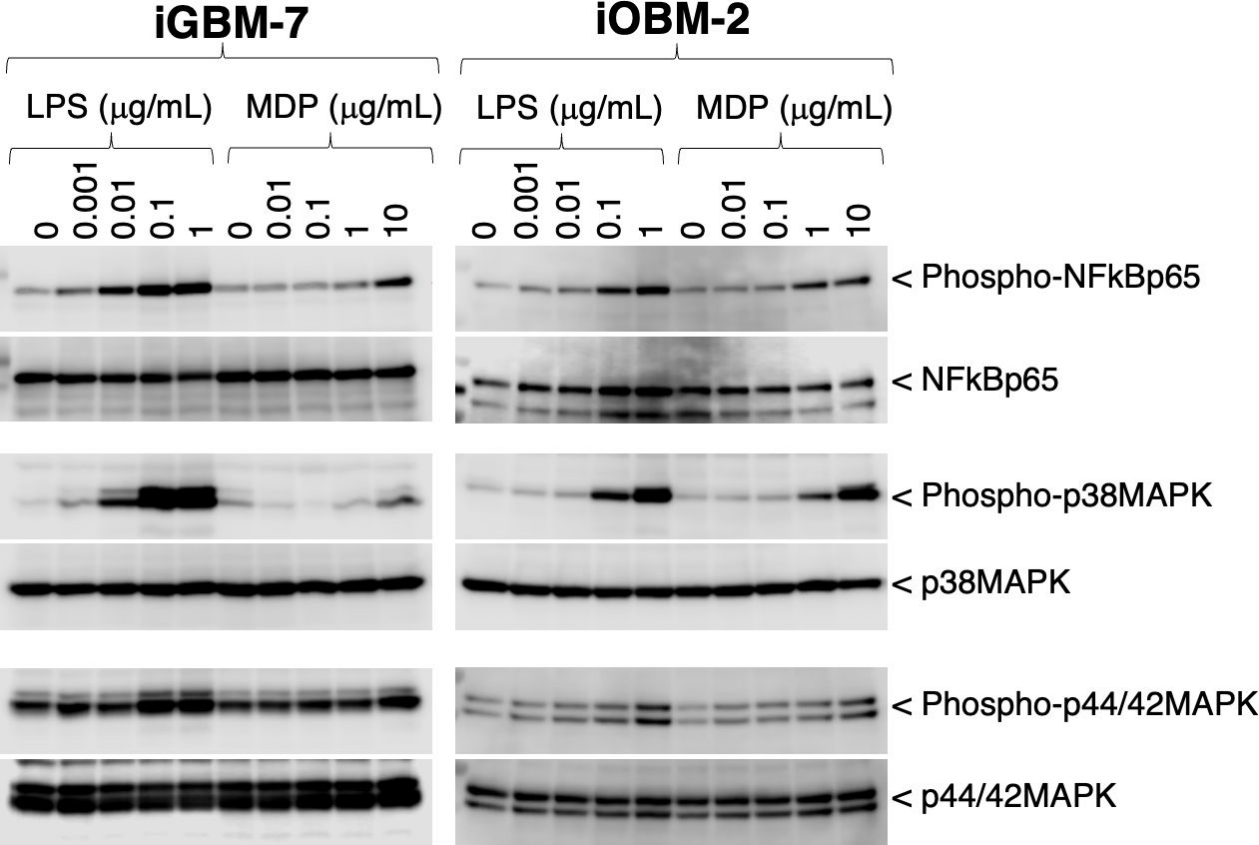
Phosphorylation of NF-kB p65, p38 MAPK, and p44/42 MAPK in iGBM-7 and iOBM-2 cells in response to LPS or MDP. A treatment with LPS or MDP induced the phosphorylation of NF-kB p65, p38 MAPK, and p44/42 MAPK in a dose-dependent manner (*first*, *third*, and *fifth panels*). The equivalent protein loading of these molecules was confirmed by immunoblotting with anti-NF-kB p65, anti-p38 MAPK, and anti-p44/42 MAPK antibodies (*second*, *fourth*, and *sixth panels*). Blots are representative of two or three independent experiments.

To evaluate phagocytotic activity, iGBM-7 and iOBM-2 cells were treated with pHrodo Green-labeled *Escherichia coli* BioParticles, and fluorescence was monitored by a flow cytometer. pHrodo Green-derived fluorescence, representing phagosome maturation, increased in a time-dependent manner in these cells (Fig. 5A and B). In addition, to evaluate the activation of cytoplasmic signaling molecules, iGBM-7 and iOBM-2 cells were stimulated with the bacterial cell wall components, LPS and muramyl dipeptide (MDP). Both stimuli induced the phosphorylation of nuclear factor-kB (NF-κB) p65, p38 mitogen-activated protein kinase (MAPK), and p44/42 MAPK in a dose-dependent manner (Fig. 6).

### Responses of iGBM-7 and iOBM-2 cells to CAEV infection

**Fig 7:**
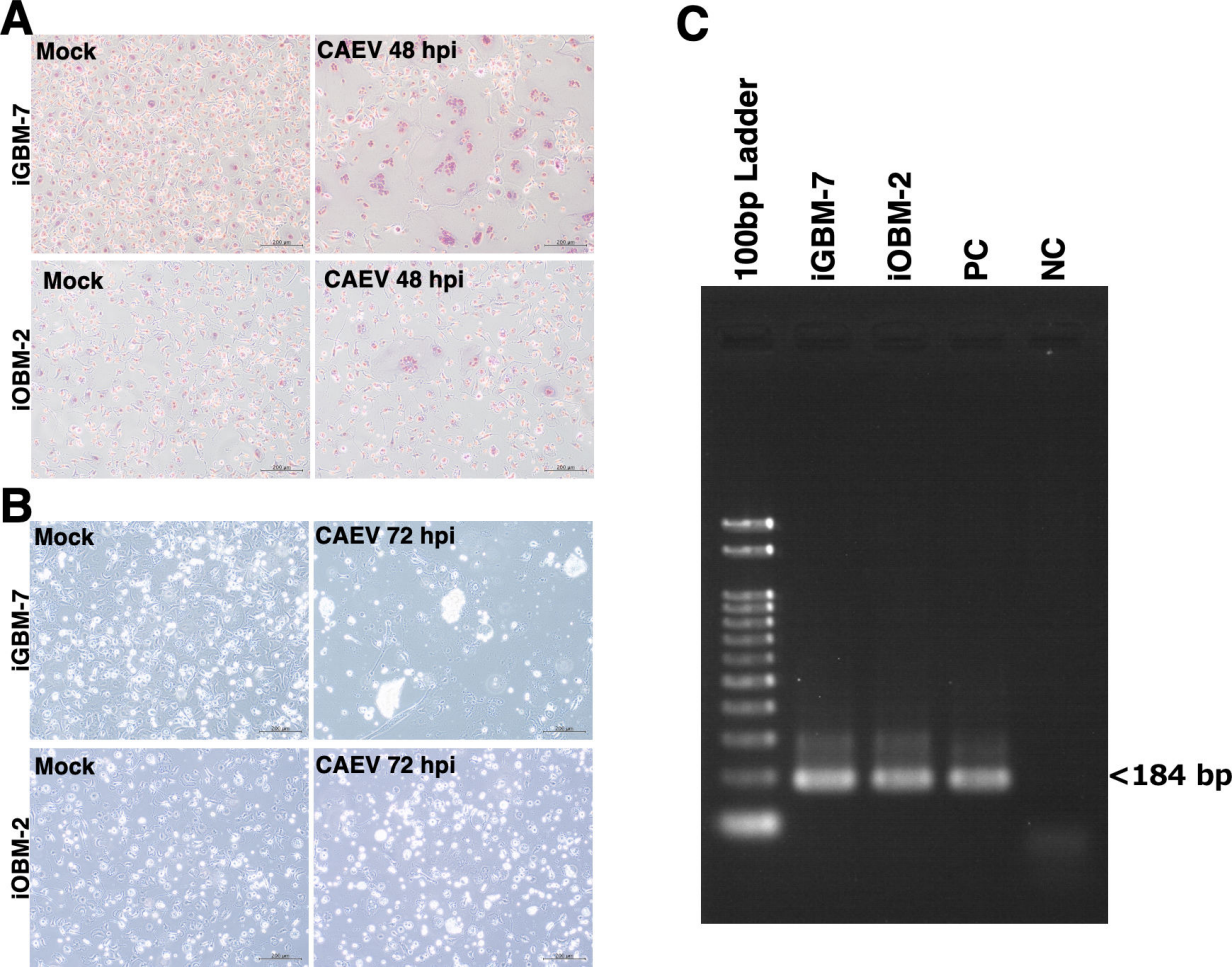
Responses of iGBM-7 and iOBM-2 cells after CAEV infection. Infection with CAEV induced the formation of MGCs at 48 hpi (**A**) and CPEs at 72 hpi (**B**) in iGBM-7 and iOBM-2 cells. Integration of the CAEV proviral genome was detected in the genomic DNAs of iGBM-7 and iOBM-2 cells (**C**). DNA samples extracted from CAEV-infected FLK cells and CAEV-uninfected FLL cells were used as the positive control (PC) and negative control (NC), respectively (**C**). The PCR products derived from CAEV proviral DNA were 184 bp in length (**C**).

The susceptibility of iGBM-7 and iOBM-2 cells to CAEV was assessed by the formation of MGCs and induction of cytopathic effects (CPEs). iGBM-7 cells infected with CAEV formed many MGCs by 48 h post-infection (hpi) (Fig. 7A) and exhibited CPEs by 72 hpi (Fig. 7B). Although CAEV-infected iOBM-2 cells formed MGCs and exhibited CPEs, these changes were less pronounced than those in iGBM-7 cells (Fig. 7A and B). The integration of the CAEV proviral genome was detected in the genomic DNA of iGBM-7 and iOBM-2 cells infected with CAEV (Fig. 7C).

### Propagation of arthropod-borne viruses (arboviruses) in an iGBM-7 or iOBM-2 cell culture

**Fig 8:**
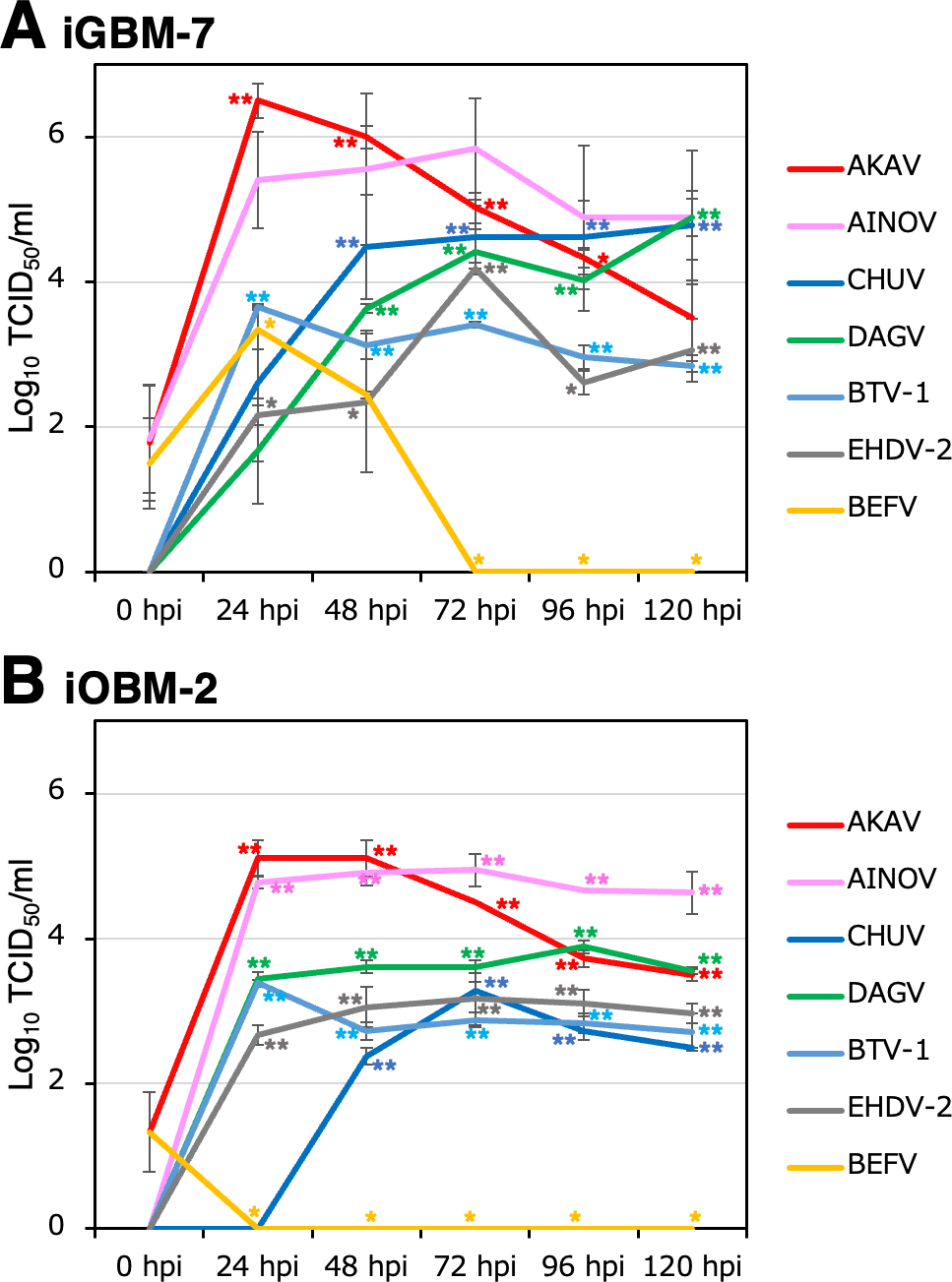
Propagation of arboviruses in iGBM-7 and iOBM-2 cell cultures. Cells were infected with the AKAV OBE-1 strain (*red line*), AINOV JaNAr28 strain (*pink line*), CHUV isolate 31 (*blue line*), the DAGV KSB-29/E/01 strain (*green line*), BTV-1 ON-24/E/17 strain (*light blue line*), EHDV-2 strain No. 2 (*gray line*), and BEFV YHL strain (*yellow line*) at MOI = 0.1. Culture supernatant samples were collected at the indicated time points post-infection. Viral production in the iGBM-7 (**A**) and iOBM-2 (**B**) cell cultures was estimated by titration experiments with HmLu-1 cells. Data represent the mean ± SEM of three independent experiments (**P < 0.01, *P < 0.05 vs. 0 hpi).

We investigated whether iGBM-7 and iOBM-2 cells were susceptible to infection with various arboviruses and also if they supported the intracellular replication of these viruses. The Akabane virus (AKAV) OBE-1 strain and Aino virus (AINOV) JaNAr28 strain efficiently propagated in both the iGBM-7 and iOBM-2 cell cultures, reaching maximum titers of 10^5^–10^6^ 50% tissue culture infectious dose (TCID_50_)/mL at 24 hpi (Fig. 8A and B). When these cells were infected with Chuzan virus (CHUV) isolate 31 and the DʼAguilar virus (DAGV) KSB-29/E/01 strain, virus growth was more efficient in iGBM-7 cell cultures than in iOBM-2 cell cultures (Fig. 8A and B). Bluetongue virus serotype-1 (BTV-1) and epizootic hemorrhagic disease virus serotype 2 (EHDV-2) moderately propagated in both cell cultures and reached maximum titers of 10^2^–10^3^ TCID_50_/mL within 120 hpi (Fig. 8A and B). iGBM-7 and iOBM-2 cells did not appear to support the propagation of bovine ephemeral fever virus (BEFV) (Fig. 8A and B).

## DISCUSSION

Novel sustainable cell lines of goat and sheep blood macrophage origins were established in the present study using the mixed cell culture technique with porcine primary kidney cells as feeder cells. The successful expansion of GBMs and OBMs using the mixed culture method suggested that primary porcine kidney cells could support the growth of small ruminant-derived macrophages, despite their origin from a different mammal. Similar to primary macrophages from other mammalian origins (10–15), GBMs and OBMs preferentially adhered to NTC-dishes and were easily separated from other cell types, such as the lymphocytes of blood and feeder cells detached from cell sheets. It is important to note that despite the immortalization protocol for porcine macrophages shown in previous studies (9, 16, 17), GBMs and OBMs were immortalized by introducing both the SV40LT and pTERT genes, and macrophage cell lines from goat and sheep origins were successfully obtained. Although this protocol has been applied to blood macrophages from red river hogs, a member of the same *Suidae* family as pigs (14), this is the first study to demonstrate that this protocol is effective for immortalizing macrophages from small ruminants.

As a characteristic of macrophages, whole iGBM-7 and iOBM-2 cells were immunocytochemically positive for Iba1, a cytoplasmic protein that plays a significant role in the activation and function of macrophages (18). Regarding cell surface markers, both cell lines were positive for CD172a, which is expressed on cells of myeloid origin and is indicative of a DC and macrophage-like phenotype (19), and CD204, known as a class A macrophage scavenger receptor (20). These findings indicate that iGBM-7 and iOBM-2 cells are of macrophage origin. The immediate induction of phagocytotic activity upon the *E. coli* BioParticles treatment also confirmed the macrophage properties of these cell lines. In addition, the treatment with LPS or MDP induced the phosphorylation of the NF-kB p65 subunit, p38 MAPK, and p44/42 MAPK. The LPS-induced activation of NF-kB was likely followed by the up-regulated expression of pro-inflammatory cytokine mRNAs, such as IL1A, IL1B, and IL6. These properties suggest that iGBM-7 and iOBM-2 cells exhibit classically activated pro-inflammatory M1 macrophage polarization in the basal state (21).

The generation of sheep macrophage cell lines has been reported in a number of studies. Spontaneously proliferating adherent mononuclear cell lines were established from sheep blood (22). However, the expression of monocyte/macrophage markers was lower in these cells than in sheep alveolar macrophages (22). Liu et al. also reported the establishment of sheep macrophage cell lines from peripheral blood adherent cells that spontaneously proliferated in a long-term culture (23). These cell lines exhibited a long spindle-shaped morphology distinct from the typical macrophage-like morphology, and the expression of proinflammatory cytokine mRNAs following treatment with LPS was lower in these cells than in alveolar and splenic macrophages (23). Other groups additionally reported the establishment of different macrophage cell lines derived from sheep blood (24) and spleen (25); however, the macrophage properties of these cell lines have yet to be fully defined, and no cell lines are yet available worldwide. In consideration of these findings, iOBM-2 cells have clear advantages as a sheep macrophage cell line, not only in their proliferative ability but also in the performance of macrophage functions and are expected to be widely distributed as a defined cell line.

To the best of our knowledge, the generation of a goat macrophage cell line has not yet been reported, suggesting that iGBM-7 cells are promising as the first cell line suitable for application to analyze the interactions between pathogens and host immune cells in goats. Bovine macrophage (BoMac) cells have been established as a ruminant macrophage cell line (26) and have been used to study the interactions of macrophages with various bovine viruses, such as bovine foamy virus (27), bovine immunodeficiency virus (28), bovine herpesvirus 4 (29, 30), and lumpy skin disease virus (31). However, evidence showing that BoMac cells are persistently infected with BVDV makes the interpretation of these findings difficult (32). We herein confirmed that iGBM-7 and iOBM cell cultures were free of BVDV contamination, a clear advantage as ruminant cell lines.

CAEV is the causative agent of caprine arthritis-encephalitis, a persistent infectious disease in goats (33). CAEV may be transmitted to sheep in farms where goats and sheep are kept together (34). The main target cells for CAEV are the monocyte/macrophage lineage (35), and viral replication appears to be restricted to M1 macrophages but increases in alternatively activated anti-inflammatory M2 macrophages (8). Since iGBM-7 and iOBM-2 cells basically exhibit the M1 phenotype, their conversion to the M2 phenotype may increase their susceptibility to CAEV infection. A treatment with dexamethasone, an anti-inflammatory steroid, may effectively induce an increase in the M2 phenotype population in iGBM-7 and iOBM-2 cells (36, 37), which may enhance their susceptibility to CAEV. These cell lines are also expected to be useful in clarifying the effects of CAEV on the innate immune function of goat or sheep macrophages.

AKAV and AINOV belong to the *Orthobunyavirus* species previously classified as the Simbu serogroup (38), whereas CHUV and DAGV belong to the *Orbivirus* species, which is commonly described as the Palyam serogroup (39). These viruses cause or have the potential to cause congenital malformations in cattle. In the present study, we found that the AKAV OBE-1 strain and AINOV JaNAr28 strain generally propagated more efficiently in iGBM-7 and iOBM-2 cell cultures than the CHUV isolate 31 and DAGV KSB-29/E/01 strain. These results suggest that AKAV and AINOV have more tropism for iGBM-7 and iOBM-2 cell cultures than the Palyam serogroup. The tropism of these viruses is consistent with previous findings from a hamster lung (HmLu)−1 cell culture, where AKAV and AINOV propagated more efficiently than CHUV and DAGV (40), which appeared to be due to the potential properties of these viruses. We also found that the efficiency of such virus production in iGBM-7 and iOBM-2 cell cultures was lower than that in HmLu-1 cell culture.

Among these viruses, AKAV is known as a teratogenic pathogen that causes abortions, stillbirths, premature births, and congenital abnormalities accompanied by arthrogryposis-hydranencephaly syndrome in cattle, sheep, and goats (41). A previous study reported that strong immunoreactivity for AKAV antigens was detected not only in the cytoplasm of neurons and nerve axons but also in vascular endothelial cells and macrophages that aggregate in the perivascular cuffing observed in the brain stem area of infected cattle (42). Based on these findings, iGBM-7 and iOBM-2 cells appear to be suitable tools not only for propagating AKAV but also for analyzing the effects of its infection on macrophage functions. In addition, AINOV and CHUV infections may be distributed in goats and sheep (43, 44), suggesting the potential of iGBM-7 and iOBM-2 cells for studying the isolation and characterization of these viruses.

BTV and EHDV are closely related arboviruses that infect ruminants and are transmitted between mammalian hosts by *Culicoides* biting midges (45, 46). BTV is the causative agent of bluetongue, an insect-transmitted viral disease of wild and domestic ruminants, which occurs most commonly in sheep and some species of wild ruminants, such as white-tailed deer, and sporadically in cattle (47). The cellular tropism of BTV includes macrophages, dendritic cells, and endothelial cells (48), supporting the present result showing that the BTV-1 ON-24/E/17 strain propagated in the iOBM-2 cell culture. On the other hand, EHDV-2, used in this study, primarily affects cattle and is known to cause Ibaraki disease (49). In contrast to BTV, previous studies demonstrated that EHDV infection did not produce clinical signs or viremia in sheep (50–52). In consideration of the propagation of EHDV-2 in iOBM-2 cells, this cell line may provide a practical model to study the molecular mechanisms underlying the weak or non-virulence of EHDV in sheep.

BEFV is the cause of bovine ephemeral fever, an acute febrile illness in cattle and water buffaloes (53). BEFV antigens may be detected in the endothelial cells and alveolar macrophages of the lungs of infected cattle (54, 55), suggesting a similar cellular tropism to BTV. However, the BEFV YHL strain did not propagate in the iGBM-7 or iOBM-2 cell culture, which may be attributed to its low tropism to these small ruminant-derived cells. These results may be related to previous findings showing that sheep experimentally infected with BEFV showed no clinical signs other than pyrexia and failed attempts to isolate the virus (53).

In conclusion, we produced new goat and sheep blood-derived macrophage cell lines using the immortalization protocol for porcine macrophages. The established cell lines retained macrophage functions, such as phagocytosis and pro-inflammatory activity. Furthermore, we verified the usefulness of these cell lines in evaluating various ruminant viruses, as summarized in Table 1. These cell lines have emerged as a practical and reliable tool to investigate the host-pathogen interactions occurring in infectious diseases in ruminants due to their high susceptibility to CAEV, which primarily affects goats, and arboviruses, which primarily affect cattle.

**TABLE 1:** Summary of virus characteristics investigated in this study

Virus (abbreviation) strain (reference)	Family genus	Primarily affected livestock	Putative target cells	Cells used for virus characterization or titration	Responses in iGBM-7 and iOBM-2 cells
Caprine arthritis encephalitis virus (CAEV) Strain isolated in Japan (56)	*Retroviridae Lentivirus*	Goat	Monocytes and macrophages	Fetal lamb lung (FLL) cells	Induction of syncytia and cytopathic effects
Akabane virus (AKAV) OBE-1 (38)	*Peribunyaviridae Orthobunyavirus*	Cattle	Neuronal cells	HmLu-1 cells	Efficient virus production
Aino virus (AINOV) JaNAr28 (38)	*Peribunyaviridae Orthobunyavirus*	Cattle	Neuronal cells	HmLu-1 cells	Efficient virus production
Chuzan virus (CHUV) isolate 31 (40)	*Sedoreoviridae* *Orbivirus*	Cattle	Neuronal cells	HmLu-1 cells	Moderate or low virus production
DʼAguilar virus (DAGV) KSB-29/E/01 (40)	*Sedoreoviridae* *Orbivirus*	Cattle	Neuronal cells	HmLu-1 cells	Moderate or low virus production
Bluetongue virus serotype-1 (BTV-1) ON-24/E/17 (45)	*Sedoreoviridae* *Orbivirus*	Sheep	Macrophages, dendritic cells, and endothelial cells	HmLu-1 cells	Moderate or low virus production
Epizootic hemorrhagic disease virus serotype 2 (EHDV-2) No. 2 (49)	*Sedoreoviridae* *Orbivirus*	Cattle	Endothelial cells	HmLu-1 cells	Moderate or low virus production
Bovine ephemeral fever virus (BEFV) YHL (53)	*Rhabdoviridae Ephemerovirus*	Cattle	Endothelial cells and macrophages	HmLu-1 cells	Less virus production

## MATERIALS AND METHODS

### Isolation of primary macrophages from the peripheral blood of goats and sheep

Based on our previous studies, macrophage-depleted porcine kidney primary cell cultures were used as feeder cells for the propagation of GBMs and OBMs (9). Feeder cells were cultured in growth medium composed of Dulbecco’s modified Eagle’s medium (DMEM) (Nacalai Tesque, Inc., Kyoto, Japan) containing 10% heat-inactivated fetal bovine serum (FUJIFILM Wako Pure Chemical Corp., Osaka, Japan) and supplemented with 25 µM monothioglycerol (FUJIFILM Wako), 10 µg/mL insulin (Sigma, St. Louis, MO), streptomycin-penicillin (100 µg/mL and 100 U/mL, respectively) (Nacalai Tesque), and 5 µg/mL Fungin (InvivoGen, San Diego, CA). Two milliliters of heparinized peripheral blood were directly added to the feeder cell culture in T-150 tissue culture flasks (Sumitomo Bakelite Co., Ltd., Tokyo, Japan) and cultured at 37°C in a humidified atmosphere of 95% air and 5% CO_2_. The culture medium was replaced every 3–4 days. After approximately 2 weeks, macrophage-like cells that loosely attached to the cell sheet appeared and were harvested from the culture supernatant after centrifugation. Similar to our previous findings (12, 16, 17, 57), GBMs and OBMs preferentially adhered to NTC-dishes (Sumitomo Bakelite) and, thus, were separated from other cell types based on this feature.

### Immortalization of GBMs and OBMs

Lentiviral particles carrying the SV40LT gene or pTERT gene were prepared as previously described (9). GBMs or OBMs seeded on 60-mm NTC-dishes were exposed to these lentiviral particles in the presence of 6 µg/mL of Polybrene (Nacalai Tesque), and iGBM or iOBM was subsequently established after 1–2 months of culture. Species identification analyses of iGBM and iOBM cells were performed at Seibutsugiken Co., Ltd. (Kanagawa, Japan).

In subcultures, iGBM or iOBM cells (1 × 10^6^) were seeded on 90-mm NTC-dishes (Sumitomo Bakelite) and continuously passaged every 5–6 days. At each passage, cells were detached using TrypLE Express Solution (Thermo Fisher Scientific, Waltham, MA), and the number of harvested cells was measured using a Bio-Rad TC20 automated cell counter.

### Immunocytochemistry

Cells were seeded on eight-well chamber slides (Asahi Glass Co., Ltd., Tokyo, Japan) at a density of 2 × 10^5^ cells/well. The next day, after being washed once with Dulbecco’s phosphate-buffered saline (DPBS), the cells were fixed using 4% paraformaldehyde phosphate buffer solution (Nacalai Tesque), permeabilized with 1% Triton X-100/phosphate-buffered saline solution, and blocked with Blocking One Histo (Nacalai Tesque). Cells were then incubated with the primary antibodies at room temperature for 1 h, and the EnVision system (DAKO, Hamburg, Germany) was used to visualize antibody-antigen reactions, according to the manufacturer’s procedure. Cell nuclei were counterstained with Mayer’s hematoxylin solution (FUJIFILM Wako), and stained slides were examined under a microscope (Leica, Bensheim, Germany).

The primary antibodies used for immunocytochemistry were as follows: mouse monoclonal antibodies against CD172a (clone DH59B) (VMRD, Inc., Pullman, WA) and CD204 (clone SRA-E5) (TransGenic, Inc., Kobe, Japan), and rabbit polyclonal antibodies against ionized calcium-binding adaptor molecule 1 (Iba1) (FUJIFILM Wako).

### PCR analysis

The successful transduction of the SV40LT and pTERT genes into the iGBM or iOBM genome was confirmed by genomic DNA PCR. PCR experiments were performed as described in our previous study (16). The lengths of PCR products derived from the SV40LT and pTERT genes were 128 and 143 base pairs (bp), respectively.

The insertion of CAEV proviral DNA (a part of the *gag* region) into the iGBM or iOBM genome was confirmed by nested PCR using two primer pairs as previously described (56). In brief, the genomic DNA of CAEV-infected cells was extracted using the DNeasy Blood & Tissue kit (QIAGEN, PL Venlo, Netherlands), and PCR experiments were performed using KOD FX DNA polymerase (TOYOBO, Osaka, Japan). In the second PCR amplification, PCR products derived from CAEV proviral DNA were 184 bp in length and analyzed with agarose gel electrophoresis using GelGreen solution (Biotium, Inc., Fremont, CA). DNA samples extracted from CAEV-infected fetal lamb kidney (FLK) cells and CAEV-uninfected fetal lamb lung (FLL) cells were used as positive and negative controls, respectively (56).

### qRT-PCR analysis of measurements of cytokine mRNA expression

Cells (4 × 10^5^ cells/well in a 24-well plate) were primed with 1 µg/mL LPS (Sigma) at 37°C for 3 h in a growth medium (400 µL). Cells were lysed, and total RNA was extracted using the NucleoSpin RNA kit (Takara Bio Inc., Shiga, Japan). Fifty nanograms of total RNA were synthesized into cDNA using the PrimeScript RT reagent Kit with gDNA Eraser (Takara Bio Inc.), and the resulting cDNA was subjected to qRT-PCR with the Thermo Scientific SYBR Green qPCR Master Mix (Thermo Fisher Scientific). Amplification was conducted on QuantStudio 3 (Thermo Fisher Scientific) under the following conditions: enzyme activation step at 95°C for 2 min, followed by 40 cycles of 95°C for 15 s (denaturation) and 60°C for 30 s (annealing and extension). Goat- or sheep-specific cytokine primer pairs were designed using Primer3Plus (https://www.primer3plus.com) and purchased from Sigma (Table 2). The relative expression of the genes of interest was estimated using the delta-delta method with normalization to the GAPDH gene as the control gene (58). No significant differences were observed in the amount of GAPDH between the groups for all the samples investigated. Two or three independent experiments were performed, and data are expressed as the mean ± standard deviation.

**TABLE 2:** Primers used for the RT-qPCR analysis

Primer	Sequence (5′ → 3′)	GenBank accession numbers (Position)
Goat IL1A sense primer	CCTGGAAGCCATTGCCAATG	D63350 (304–323)
Goat IL1A antisense primer	GCGTCGTTCAGGATGCATTC	D63350 (432–413)
Goat IL1B sense primer	ATGAGCTTCTGTGTGACGCA	D63351 (329–348)
Goat IL1B antisense primer	GAACACCACTTCTCGGCTCA	D63351 (471–452)
Goat IL6 sense primer	CCACTGCTGGTCTTCTGGAG	NM_001285640 (344–363)
Goat IL6 antisense primer	TGTTTGTGGCTGGAGTGGTT	NM_001285640 (505–486)
Goat GAPDH sense primer	ATCAAGTGGGGTGATGCTGG	AJ431207 (151–170)
Goat GAPDH antisense primer	GGTTCACGCCCATCACAAAC	AJ431207 (307–288)
Sheep IL1A sense primer	CCTGGAAGCCATTGCCAATG	NM_001009808 (288–307)
Sheep IL1A antisense primer	GCGTCGTTCAGGATGCATTC	NM_001009808 (416–397)
Sheep IL1B sense primer	ACAGATGAAGAGCTGCACCC	NM_001009465 (119–138)
Sheep IL1B antisense primer	CTGCCTGCCTGAAGCTTTTG	NM_001009465 (225–206)
Sheep IL6 sense primer	TGCAGTCCTCAAACGAGTGG	NM_001009392 (570–589)
Sheep IL6 antisense primer	CCGCAGCTACTTCATCCGA	NM_001009392 (679–661)
Sheep GAPDH sense primer	ACAGTCAAGGCAGAGAACGG	NM_001190390 (208–227)
Sheep GAPDH antisense primer	AGTGAAGACCCCAGTGGACT	NM_001190390 (342–323)

### Phagocytotic assay using pHrodo-labeled *E. coli* BioParticles

Cells (5 × 10^5^) were cultured with growth medium in 35-mm NTC dishes. The next day, cells were treated with or without 20 µg/mL of pHrodo Green dye-conjugated *E. coli* BioParticles (Thermo Fisher Scientific). After 5-min, 1-h, and 4-h incubations, cells were detached using TrypLE Express Solution, re-suspended in DPBS, and then subjected to the analysis using the BD Accuri C6 Plus flow cytometer (BD Biosciences). The fluorescence of 40,000 cells was assessed in each experiment. Three independent experiments were performed, and mean fluorescence intensity (MFI) data were expressed as the mean ± standard error of the mean (SEM). Mean values were analyzed with a one-way analysis of variance (ANOVA), followed by Dunnett’s post-hoc test using the software GraphPad InStat 3 for Windows. The significance of differences was set at *P* < 0.05.

### Immunoblotting

Cells (4 ×1 0^5^ cells/well in a 24-well plate) were stimulated with LPS or MDP (InvivoGen) in serum-free DMEM at the concentrations indicated. After being incubated at 37°C for 30 min, culture supernatants were collected, and cells were lysed with 100 µL ice-cold lysis buffer (50 mM Tris-HCl [pH 7.4], 150 mM NaCl, 0.5% Triton X-100, and 0.5% sodium deoxycholate) containing cOmplete mini protease inhibitor (Roche Diagnostics GmbH, Mannheim, Germany) and PhosSTOP tablets (Roche). Equal volumes of the cell lysate (25 µL) were separated by sodium dodecyl sulfate-polyacrylamide gel electrophoresis and electroblotted onto polyvinylidene difluoride membranes (Merck Millipore Ltd., Carrigtwohill, Ireland). The membranes were then incubated with primary antibodies, before being incubated with horseradish peroxidase-conjugated secondary antibodies. The target proteins were revealed using Chemi-Lumi One ultra (Nacalai Tesque) and detected using a C-DiGit blot scanner (LI-COR, Inc., Lincoln, NE).

The primary antibodies used for immunoblotting were as follows: rabbit monoclonal antibodies against phospho-p38 MAPK (D3F9, Cat. No. #4511), p38 MAPK (D13E1, Cat. No. #8690), phospho-NF-κB p65 (93H1, Cat. No. #3033), and phospho-p44/42 MAPK (D13.14.4E, Cat. No. #4370) (Cell Signaling Technology, Inc., Danvers, MA); rabbit polyclonal antibodies against NF-κB p65 (Cat. No. #3034) and p44/42 MAPK (Cat. No. #9102) (Cell Signaling Technology).

### CAEV infection

Cells were seeded at 4 × 10^5^/well on six-well tissue culture plates (Sumitomo Bakelite). The next day, after being washed three times with Earl’s balanced salt solution (EBSS), cells were inoculated with 200 µL of CAEV solution. CAEV stock solutions prepared from field samples (viral titers not assessed) were used in the present study (56). After the cells had been cultured for 48 h, they were washed three times with PBS, fixed with methanol, and stained with Giemsa solution (Sigma). Stained cells were observed under a microscope (Olympus, Tokyo, Japan).

### Arbovirus growth assay

Cells were seeded at 1 × 10^5^/well on 24-well tissue culture plates (Sumitomo Bakelite). The next day, after being washed three times with EBSS, cells were inoculated with arboviruses at a multiplicity of infection (MOI) of 0.1. MOI was calculated using the TCID_50_ values of each virus stock. The arboviruses used were as follows: the AKAV OBE-1 strain, AINOV JaNAr28 strain, CHUV isolate 31, the DAGV KSB-29/E/01 strain, BTV-1 ON-24/E/17 strain, EHDV-2 strain No. 2, and BEFV YHL strain. After cells had been incubated at 37°C for 1 h, the inoculum was removed, and cells were washed three times with EBSS. Growth medium containing 2% FBS was then added, and culture supernatants were collected at 0, 24, 48, 72, 96, and 120 hpi. Virus titrations were analyzed using CPEs against HmLu-1 cells as described in our previous study (40). Viral titers were expressed as TCID_50_/mL. The viral growth assay was independently performed three times for each arbovirus, and data are expressed as the mean ± SEM. Mean values were analyzed with a one-way ANOVA followed by Dunnett’s post-hoc test using the software GraphPad InStat 3 for Windows. The significance of differences was set at *P* < 0.05.
